# Zoledronic acid is more efficient than ibandronic acid in the treatment of symptomatic bone marrow lesions of the knee

**DOI:** 10.1007/s00167-019-05598-w

**Published:** 2019-07-04

**Authors:** Fabio Müller, Konrad A. Appelt, Christian Meier, Norbert Suhm

**Affiliations:** 1 Department of Orthopaedics and Traumatology, University Hospital Basel, Spitalstrasse 21, 4031 Basel, Switzerland; 2 Department of Radiology, University Hospital Basel, Basel, Switzerland; 3 Department of Endocrinology, Diabetes and Metabolism, University Hospital Basel, Basel, Switzerland; 4 ENDONET, Basel, Switzerland

**Keywords:** Bone marrow lesion, Bone marrow oedema, Transient osteoporosis, Bone bruise, Bisphosphonates, Biphosphonate, Antiresorptive medication, Bone turnover marker, Magnetic resonance imaging (MRI)

## Abstract

**Purpose:**

The purpose of this study was to determine the efficacy and tolerability of different antiresorptive therapeutic regimens for treating symptomatic bone marrow lesions (BML) of the knee.

**Methods:**

Patient records of 34 patients with radiologically diagnosed, painful BML of the knee treated with either a bisphosphonate (zoledronic, ibandronic, or alendronic acid) or with a human monoclonal antibody (denosumab) were retrospectively evaluated. Response to treatment was assessed, as change in patient-reported pain, by evaluation of BML expansion on MRI using the Whole-Organ Magnetic Resonance Imaging Score (WORMS), and by laboratory analysis of bone turnover markers: C-terminal cross-linking telopeptide (CTx) and procollagen type 1 amino-terminal propeptide (P1NP). Tolerability was evaluated by documentation of adverse reactions.

**Results:**

Zoledronic acid was more or at least equally effective as the other treatment regimens with response to treatment in 11 of 12 patients (92%). The highest rate of adverse events was noted in 4 of 12 patients (33%) treated with zoledronic acid. CTx and WORMS differentiated well between responders and non-responders, whereas P1NP failed to do so. Changes in pain correlated moderately with change in WORMS (*r* = − 0.32), weakly with change in CTx (*r* = − 0.07), and not at all with change in P1NP.

**Conclusion:**

Zoledronic acid appeared to be more effective than other antiresorptive medications—at the cost of more frequent adverse events. While radiological and laboratory evaluation methods may allow for objective treatment monitoring, they appear to capture different dimensions than patient-reported pain.

**Level of evidence:**

III.

## Introduction

**Fig. 1:**
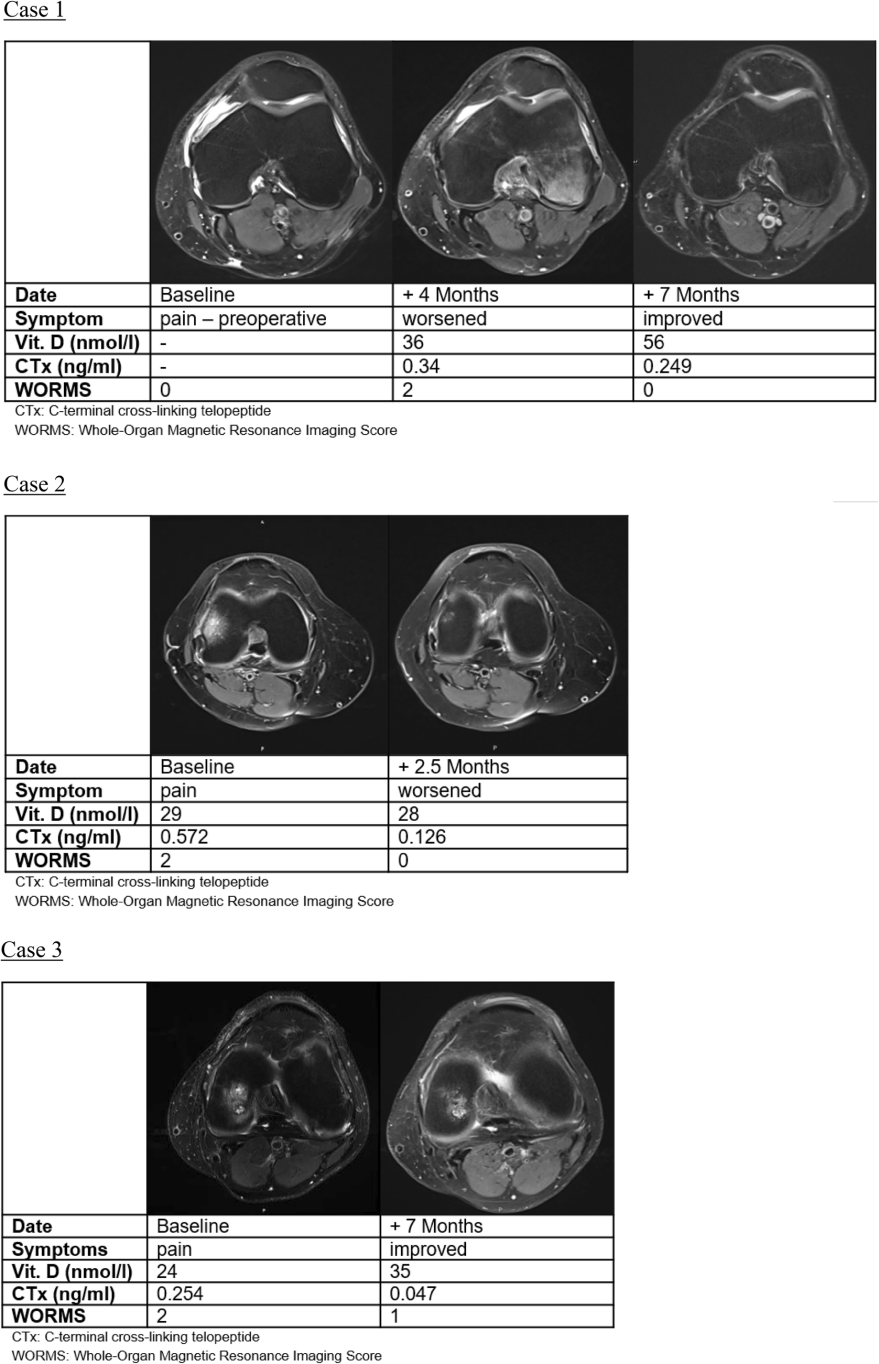
Clinical, radiological, and laboratory course of BML cases: in the following, presentation of imaging planes is restricted to axial orientation due to limited space. Case 1: BML after surgery, spontaneous course: MRI was ordered post-operatively because of increasing pain. Spontaneous course, without antiresorptive treatment. Note the concordance of clinical, radiological and laboratory course. Case 2: primary BML, treatment failure: female patient, 57 years old, without a history of relevant trauma, no previous surgery. MRI was ordered because of unsatisfactory clinical course following treatment with a single administration of ibandronic acid. Note the discordance of clinical with radiological and laboratory course. Case 3: traumatic BML, successful treatment: the MRI at baseline was ordered due to the unsatisfactory clinical course after surgery. Treatment with a single administration of ibandronic acid. Note the concordance of clinical, radiological, and laboratory course

Bone marrow lesions (BML) are frequently observed in magnetic resonance imaging (MRI) of the knee for various pathologic conditions [3], yet their clinical significance and the need for treatment are often unclear (Fig. 1). Depending on the related pathology, BML are accompanied by the risk of progression to osteonecrosis. The previous laboratory studies revealed an altered bone metabolism in patients suffering from BML [3, 12, 16, 18]. Specifically, elevated levels of serum bone turnover markers including C-terminal cross-linking telopeptide (CTx, a bone resorption marker) and procollagen type 1 amino-terminal propeptide (P1NP, a bone formation marker) [21], as well as vitamin D deficiencies have been reported [1, 5, 19, 20]. MR imaging has been combined with bone metabolic workup to get a better understanding of the aetiopathological processes underlying BML [9, 12], and to start an indicated treatment as early as possible.

Antiresorptive medications inhibit bone resorption and are used in metabolic bone diseases characterised by increased osteoclastic activity [8, 14, 15, 18]. Using antiresorptive medication in the treatment of osteonecrosis is based on the assumption that a structural bone failure is caused by resorption of necrotic bone before the new bone has been formed. Suppressing accelerated bone resorption with antiresorptive medications until sufficient new bone has been formed could potentially avoid structural failure. However, basing treatment decision on this consideration often delays the start of the treatment because of the time needed to observe disease progression. In contrast, BML can also be a significant source of pain and antiresorptive medications are known for an analgesic effect on bone pain [11]. Hence, bone pain could be considered for determining the necessity of treating BML [18]. Moreover, vitamin D deficiencies have to be looked upon as risk factors for the development of BML [1, 5, 19, 20], and also for adverse events due to antiresorptive treatment [2]. Antiresorptive agents and vitamin D supplementation should, therefore, be co-administered.

The aim of this retrospective analysis was to investigate if symptomatic BML of the knee can be treated successfully by antiresorptive medication and vitamin D supplementation. It was hypothesised that zoledronic acid is more effective than other antiresorptive medications. The primary objective was to evaluate response to treatment by change in patient-reported pain. Combination with radiological follow-up of BML using the semiquantitative Whole-Organ Magnetic Resonance Imaging Score (WORMS) and that with laboratory analysis of bone metabolic workup using CTx and P1NP were alternatively applied for treatment monitoring. The secondary objective was to determine the suitability of these methods for objective monitoring of antiresorptive treatment. It was expected that laboratory analyses could supplement radiological follow-up by MRI. In addition, the effectiveness of vitamin D supplementation and expected normalised 25-OH-vitamin-D3 serum levels after treatment were also assessed.

## Materials and methods

This retrospective, open-label and observational analysis is part of an ongoing quality assurance measure managed by the University Hospital Basel initiated in April 2017. The analysis was approved by the regional ethics committee (EKNZ no. 2017-00338) and followed applicable law, the principles of good clinical practise, and the Declaration of Helsinki.

### Patient sample

**Fig. 2:**
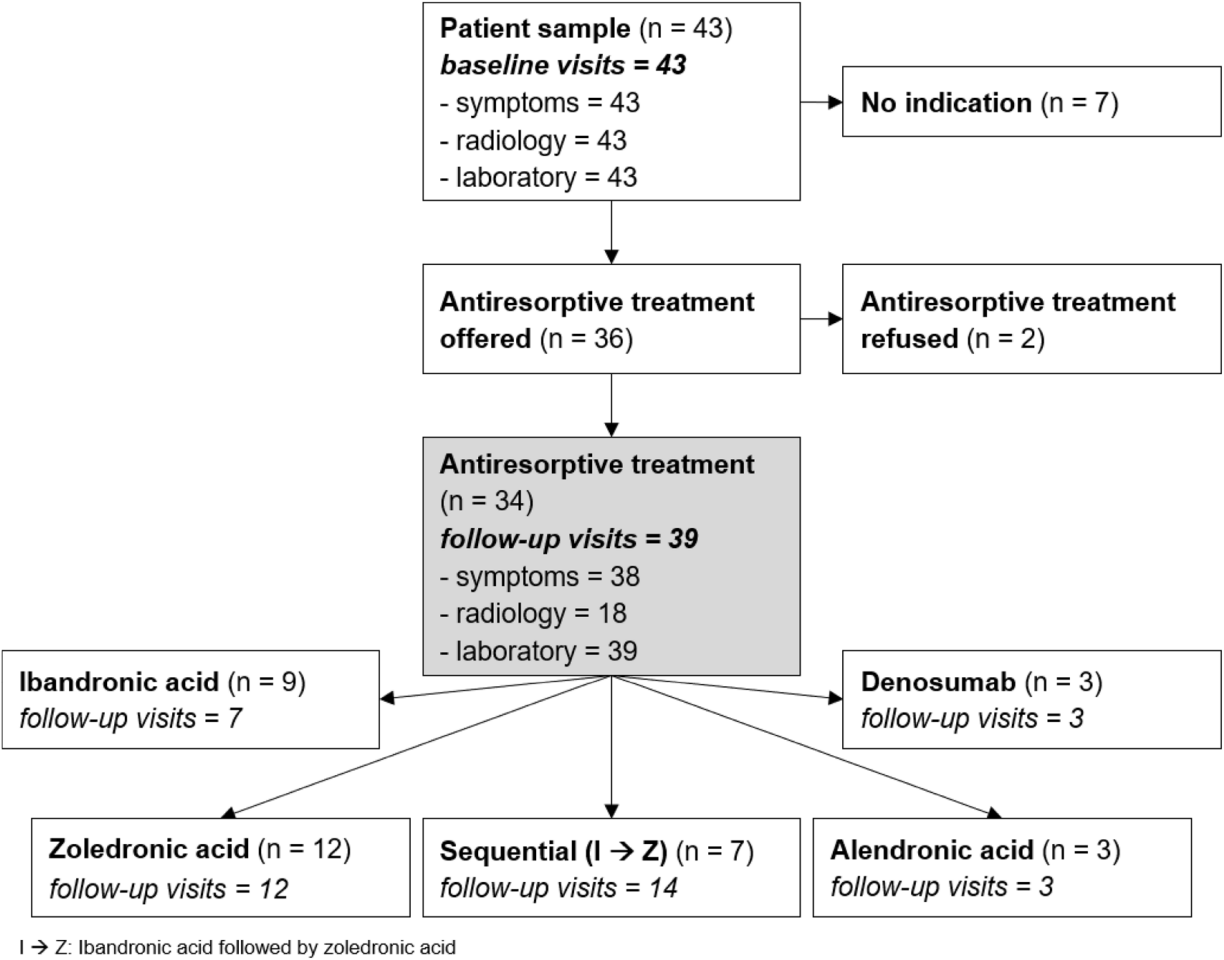
Diagnostic and therapeutic flowchart: “no indication” represents all patients not receiving antiresorptive treatment due to medical reasons, whereas “antiresorptive treatment refused” refers to patients personally refusing. Note: the number of “follow-up-visits” reflects all evaluations performed during and after antiresorptive treatment. Frequency of each diagnostic method (“symptoms” = pain, “radiology” = WORMS, and “laboratory” = CTx) performed during the visits is listed

At an osteological outpatient clinic, 43 patients (27 male; mean age 45 ± 15 years) with symptomatic BML of the knee radiologically diagnosed by means of fluid-sensitive MRI sequences were retrospectively identified. Patients were referred from three sports clinics during a 5-year observational period (February 2012–September 2017). Patients were included independent of potential aetiology, duration of symptoms, or final decision with respect to pharmaceutical treatment options. The same osteological consultant (N. S.) had personally evaluated all patients. Thirty-four patients were treated with antiresorptive medication. An overview of treatment options applied, follow-up visits, and examinations performed is given in Fig. 2.

### Data extraction

All data were extracted from patient records not specifically filed for scientific purposes. Records available both at the osteological outpatient clinic and at referring institutions were included. Initial examinations at referring institutions and laboratory analysis performed at the osteological outpatient clinic prior to treatment are named baseline visits for this analysis. Subsequent visits during/or at the end of treatment, performed at the osteological outpatient clinic or at referring institutions, are named follow-up visits throughout the article. Appraisal of the course of symptoms and laboratory follow-up were routinely conducted 4 weeks after application of antiresorptive medication or after treatment was ended. In case of persisting pain or dissatisfying course of treatment, multiple follow-up visits were necessary. In some cases, follow-up MRI examinations were ordered by referring physicians, particularly in cases of suspected aggravation or lack of pain reduction, but also for follow-up to assess response to treatment. Because the patient reporting on adverse events was potentially subjective, these were documented but not quantified.

#### Clinical data

At baseline, all 43 patients reported pain as their predominant symptom. Thirty-eight clinical follow-up visits were recorded for the 34 patients treated. At each follow-up visit, the course of pain was rated as improved, unchanged, or worsened. To evaluate the appearance of adverse events (yes/no), patient records were screened for reports on typical symptoms of an acute phase reaction such as flu-like symptoms, myalgia, exhaustion, or fever.

#### Radiological data

MRI examinations were available for all patients at baseline and for 18 patients during follow-up. Central reassessment was performed by one trained musculoskeletal radiologist (K. A.) who was presented the MRI data in random order without patient identifiers or date. Thus, the radiologist was blinded to clinical and laboratory data as well as to the course of MRI examinations.

**Fig. 3:**
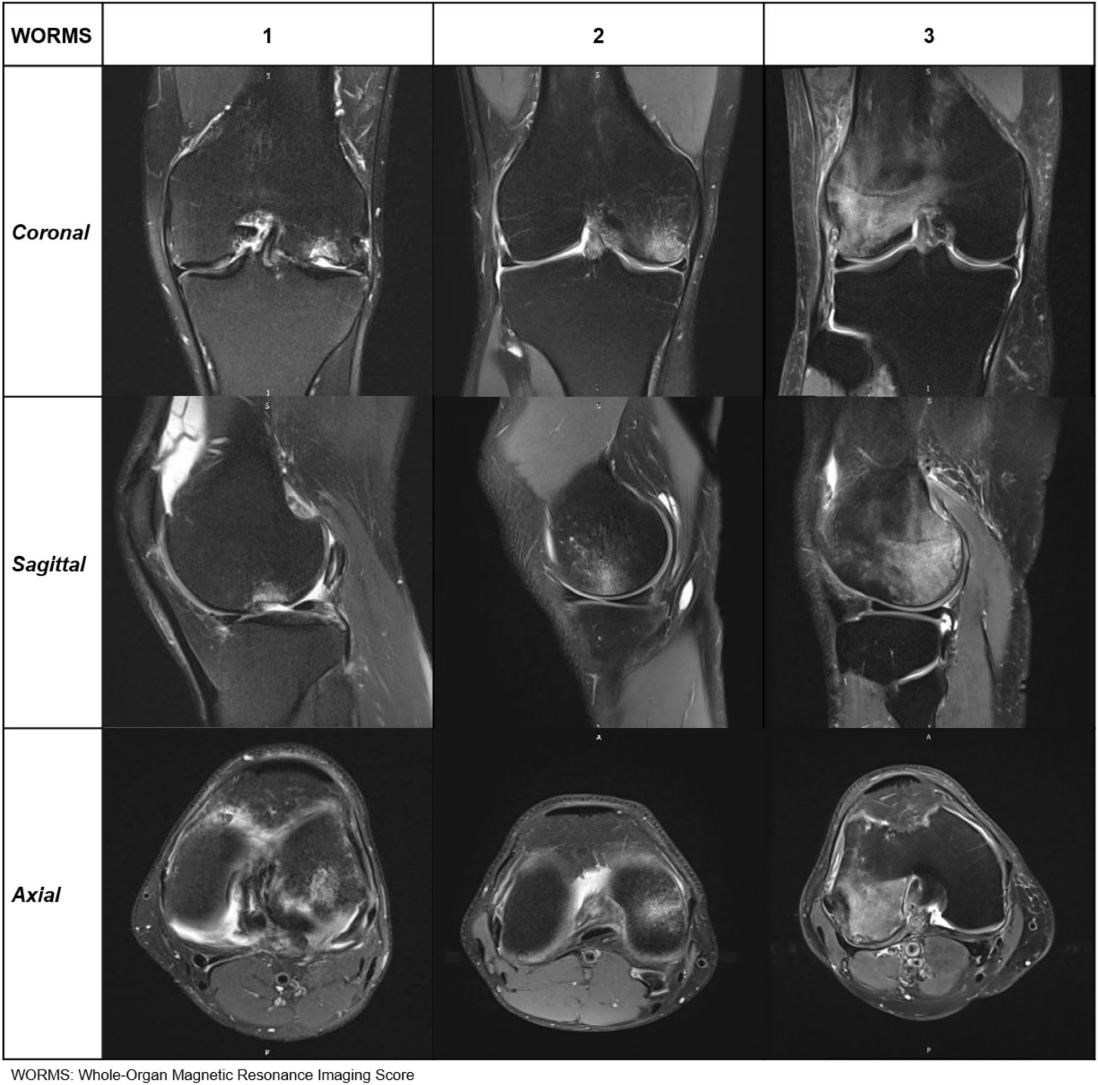
Atlas of WORMS scorings: examples on WORMS scorings 1–3 in three imaging planes. Note: WORMS 0 with no visible BML does not require presentation here

Currently, there is no standardised evaluation method for quantifying and comparing BML in MR images. The Whole-Organ Magnetic Resonance Imaging Score (WORMS) was applied to describe and quantify BML [13]. WORMS represents a semiquantitative evaluation method originally developed for research purposes in cartilage evaluation of osteoarthritic knees. The knee was divided into different compartments. The lesion volume was evaluated by the percentage of the compartment’s affected volume and scored from 0 to 3 (0 = none, 1 = affected volume < 25%, 2 = 25% ≤ affected volume < 50%, and 3 = affected volume ≥ 50%). Figure 3 illustrates examples of WORMS scorings 1–3.

#### Laboratory data

At baseline, all patients underwent laboratory analysis of bone metabolism. During follow-up, 39 laboratory analyses were performed at the osteological outpatient clinic under well-defined, standardised conditions (fasting, between 08.00 a.m. and 10.00 a.m.). In each laboratory analysis, creatinine for calculating glomerular filtration rate (GFR), 25-OH-vitamin-D3, albumin-corrected calcium in serum, CTx, and P1NP were extracted. Reference values of these laboratory parameters are presented in Table 1. Daily calcium intake by regular meals and current medication—including any type of supplements—was obtained by a standardised questionnaire and documented to complete the bone metabolic workup. CTx proved to be more sensitive than P1NP and was, thus, used as bone turnover marker in the analysis.

**Table 1:** Bone metabolic workup

Parameter	Reference range	Patients (%) in range	Patients (%) out of range		Patients unknown
GFR	> 90 ml/min	29 (74.4)	10 (25.6)		4
25-OH-vitamin-D3 baseline	75–180 nmol/l	4 (9.5)	38 (90.5)		1
25-OH-vitamin-D3 follow-up	75–180 nmol/l	6 (27.3)	16 (72.7)		12
Calcium intake	> 1200 mg/d	9 (32.1)	19 (67.9)		15
Corr. calcium baseline	2.15–2.62 mmol/l	37 (86.0)	6 (14.0)		0
Corr. calcium follow-up	2.15–2.62 mmol/l	15 (68.2)	7 (31.8)		12
Bone turnover marker			Above	Below	
CTx baseline	0.13–0.46 ng/ml	23 (53.5)	14 (32.6)	6 (13.9)	0
CTx follow-up	0.13–0.46 ng/ml	2 (8.7)	0 (0.0)	21 (91.3)	11
P1NP baseline	20–100 ng/ml	39 (90.7)	3 (7.0)	1 (2.3)	0
P1NP follow-up	20–100 ng/ml	17 (77.3)	1 (4.5)	4 (18.2)	12

Parameters and results of laboratory analyses performed at “baseline” (*n *= 43) and during “follow-up” of antiresorptive treatment (*n *= 23) are given in absolute and relative values. Examinations that were not conducted are reported by absolute values only (“unknown”)

*GFR* glomerular filtration rate, *CTx* C-terminal cross-linking telopeptide, *P1NP* procollagen 1 amino-terminal propeptide

### Decision on antiresorptive treatment indication

According to the guidelines of the osteological outpatient clinic, an indication for antiresorptive treatment initiation was based on the following criteria:

- MRI confirmed diagnosis of BML;
- symptom burden of the patient (pain, binary yes or no).

### Analytic strategy

Three different aspects (response to treatment, methods at baseline, and methods during follow-up) were analysed for this article:

#### Response to treatment

The primary outcome was change of patient-reported pain. Alternatively, the combination of pain relief, reduction in WORMS, and reduction in CTx levels of at least 50% was used to document response to treatment.

#### Diagnostic methods at baseline

To assess if laboratory analyses could replace or supplement semiquantitative evaluation of BML expansion on MRI using WORMS, correlations between WORMS and CTx were calculated at baseline. Levels for 25-OH-vitamin-D3 were measured at baseline.

#### Diagnostic methods during follow-up

To assess a diagnostic technique’s potential in treatment monitoring, the respective values of WORMS and CTx or P1NP at baseline and during follow-up were compared. Correlations between these methods were calculated for values during follow-up. To investigate the potential of laboratory analysis and/or MRI to objectively support the patient-reported primary outcome, correlations between changes in pain, WORMS, and CTx were calculated, as well. Levels for 25-OH-vitamin-D3 were measured during follow-up.

### Treatment groups

Prior to the implementation of antiresorptive treatment, all patients received vitamin D supplementation (1000 IU/d). In patients with insufficient vitamin D levels at baseline, a loading dose (45,000 IU) was also administered. Five treatment groups were considered:

- Patients treated with intravenous ibandronic acid only, multiple applications possible, and, therefore, resulting in multiple follow-up visits.
- Patients treated with intravenous zoledronic acid once only.
- Patients initially treated with intravenous ibandronic acid followed by one single administration of intravenous zoledronic acid. This group is called sequential (I → Z) in the following. Due to multiple applications of ibandronic acid and the change in medication, multiple follow-up visits possible.
- Patients treated with subcutaneous administration of denosumab once only.
- Patients treated with oral alendronic acid only.

### Statistical analysis

The distribution of binary and categorical variables was described by absolute and relative frequencies. Median, mean, or range was used to describe the distribution of continuous variables. Wilcoxon and the Kruskal–Wallis tests were used to compare the distribution of ordinal and continuous variables between groups. The Pearson correlation coefficient was used to assess the association between variables. Some patients showed BML on both femoral as well as on tibial compartments of the knee. For this analysis, WORMS was applied to the more severely affected compartment. Hence, one value for WORMS for each patient was entered into the analysis. The change from baseline was quantified by the relative change expressed in per cent. Because of the retrospective nature of the study, no formal a priori sample size calculation was performed.

## Results

### Response to treatment

The primary outcome assessed the failure of treatment based on clinical reporting of pain, or alternatively on all three methods together (“Overall”). Zoledronic acid was found to be more or at least equally effective compared to other treatment regimens (Table 2). Among the 12 patients treated with zoledronic acid, one failure due to the insufficient reduction of WORMS was noted. In all other treatment groups except for alendronic acid, higher failure rates based on missing pain relief or lack of reduction in CTx were observed. The highest rate of adverse events was noted in patients with zoledronic acid (Table 2).

**Table 2:** Response to treatment

Failure	*N*	Clinic (%)	Overall (%)	Adverse events (%)
Ibandronic acid	9	1/9 (11)	3/9 (33)	1/9 (11)
Zoledronic acid	12	0/12 (0)	1/12 (8)	4/12 (33)
Sequential (I → Z)	7	1/7 (14)	1/7 (14)	1/7 (14)
Denosumab	3	0/3 (0)	1/3 (33)	0/3 (0)
Alendronic acid	3	0/3 (0)	0/3 (0)	0/3 (0)
TOTAL	34	2/34 (6)	6/34 (18)	6/34 (18)

Response to treatment according to patient-reported pain (“Clinic”) is opposed to comprehensive “Overall” judgement

*I → Z* ibandronic acid followed by zoledronic acid

### Diagnostic methods at baseline

Very low correlations between WORMS and CTx (*r* = 0.13) were noted (Table 3a). With a mean value of 37.3 nmol/l, vitamin D levels were mostly found to be below reference (Table 1).

**Table 3:** Pairwise Pearson correlations

(a) Baseline findings	WORMS
CTx	0.13

(b) Findings during follow-up	Pain	CTx
CTx	∆ − 0.13	
WORMS	∆ − 0.32	0.48 | ∆ 0.14

Pairwise Pearson correlations for absolute values [baseline findings (3a), findings during follow-up (3b)]. Relative values [changes from baseline (3b), highlighted with the prefix “∆”]

*CTx* C-terminal cross-linking telopeptide, *WORMS* Whole-Organ Magnetic Resonance Imaging Score

### Diagnostic methods during follow-up

**Fig. 4:**
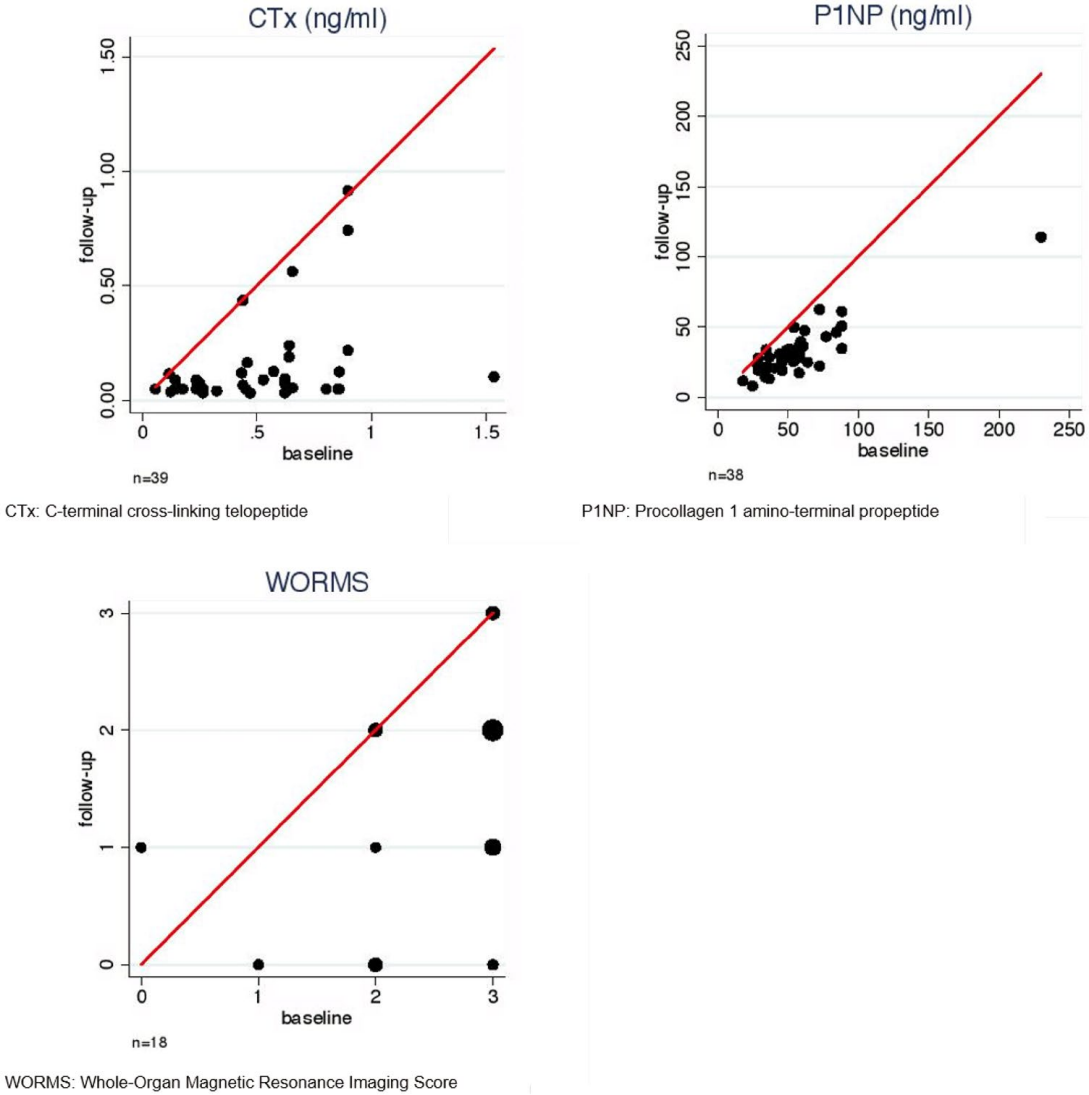
Potential of diagnostic parameters for treatment monitoring: follow-up measurements (*y-*axis) contrasted with baseline measurements (*x-*axis) per diagnostic parameter. The red line indicates equality of a parameter when measured in the course of treatment. With expected suppression of CTx, P1NP, and WORMS during the course, positive treatment response is represented by dots below the red line

Moderate correlations between WORMS and CTx (*r* = 0.48) were noted (Table 3b). In Fig. 4 a diagnostic method’s potential to illustrate treatment response is presented. WORMS and CTx differentiated well between responders and non-responders, whereas P1NP was not able to do so. Moderate correlations between changes in pain and WORMS (*r* = − 0.32), but only little correlation between changes in pain and CTx (*r* = − 0.13) were observed. Little correlations between changes in WORMS and CTx (*r* = 0.14) were noted as well. During follow-up, mean levels of vitamin D were elevated to 57.3 nmol/l (Table 1).

## Discussion

The most important finding was that zoledronic acid was found to be more effective than ibandronic acid. Furthermore, semiquantitative evaluation of BML expansion on MRI using WORMS and assessment of bone resorption by the marker CTx were identified as potential parameters for objective treatment monitoring.

### Antiresorptive treatment

Several studies have considered BML as a preamble to later development of osteonecrosis. Different antiresorptive medications have been applied to treat BML and osteonecrosis as a possible endpoint, because antiresorptive medications inhibit bone resorption [10, 18]. Mixed results have been reported for treatment with ibandronic acid [10, 18], which may be explained by its limited capacity in suppressing bone resorption [6, 17]. In contrast, zoledronic acid features a higher antiresorptive capacity. In agreement with the above-presented results, previous studies have reported an advantage of zoledronic acid above other antiresorptive medications in inhibiting bone specific pain and in suppressing bone resorption [3, 4, 8].

### Vitamin D supplementation

Simon et al. [18] have shown that balanced vitamin D homeostasis is essential prior to the administration of antiresorptive treatment. Also in line with the above-presented findings, most patients suffering from BML who had not recently undergone vitamin D supplementation exhibited vitamin D insufficiency or even deficiency [12, 18]. Contrary to the other reports [18], reconstitution of sufficient 25-OH-vitamin-D3 levels was more difficult than expected in this study’s patient population. The finding of incomplete normalisation is supported by Oehler et al. [12]. 58% of their patient population had insufficient or even deficient levels of vitamin D despite ongoing supplementation [12]. From an osteological point of view, the combined application of vitamin D supplementation and antiresorptive medication is, therefore, mandatory.

### Correlations

Response to treatment of symptomatic BML with antiresorptive medications according to this study’s indication consequently would have to be monitored with adapted tools. It was hypothesised that methods for monitoring response to treatment were interchangeable. However, this analysis failed to prove significant correlations amongst the diagnostic techniques applied. This result is in line with the current literature reporting on difficulties to establish a correlation between diagnosed BML, pain, and MRI findings [7, 9]. An explanation for these difficulties may be differences in the dynamics when the diagnostic techniques are applied. For instance, Simon et al. [18] found that the intensity of BML and/or expansion on MRI persist over a longer period of time, even when patients were already relieved from pain. Furthermore, bone turnover markers are known for different dynamics under treatment: the bone resorption marker CTx is suppressed much faster than the bone formation marker P1NP [21]. Hence, the time gaps between the onset of symptoms, diagnostic techniques performed at baseline or during follow-up, and the implementation of medication treatment may be relevant with respect to the correlations found [18].

### Evaluation of change in pain without grading

In contrast to the previous studies [10, 16, 18], scorings such as the visual analogue scale (VAS) to objectively report on pain at baseline or to quantify changes during follow-up were not applied. Patients suffering from symptomatic BML of the knee report on pain as a complex symptom emphasising the existence of two qualities. First, they describe a sharp, intense pain that may be triggered by physical examination as well as by unintended movements. Second, they experience a deep, numb continuous pain at lower level. Because of this clinical observation, it seemed inappropriate to quantify “pain” with just a number. Moreover, patients sense pain to be modulated by activity level or by the consumption of analgesic medication. Therefore, the patient’s overall judgement of pain as “improved”, “unchanged” or “worse” was favoured over VAS values.

This analysis was based on a retrospective design without a control group. The spontaneous course of BML, therefore, still necessitates clarification. Patients were referred from three sports clinics, thereby causing inconsistencies with respect to the quality of external documentation, type of pre-treatment, and possible etiopathology of BML. There were no complete follow-up data for all patients. This was mainly caused by lacking patients’ compliance to attend planned follow-up examinations. Despite the limited sample size in our retrospective study, these results are relevant, because they suggest that when zoledronic acid is considered for therapy in a case of symptomatic BML, the patient needs to be informed about the balance between effectiveness and risk of adverse events.

## Conclusion

Zoledronic acid appeared to be more effective than other antiresorptive medications, especially when compared to ibandronic acid. More adverse events were observed with Zoledronic acid, too. Radiological and laboratory methods may allow for objective monitoring of response to antiresorptive treatment of BML. Finally, sufficient vitamin D supplementation should be ascertained in BML patients prior to treatment.

## Abbreviations

BML: Bone marrow lesion
CTx: C-terminal cross-linking telopeptide
GFR: Glomerular filtration rate
MRI: Magnetic resonance imaging
P1NP: Procollagen type 1 amino-terminal propeptide
VAS: Visual analogue scale
WORMS: Whole-organ magnetic resonance imaging score

## References

[1] Berger CE, Kröner AH, Minai-Pour MB, Ogris E, Engel A (2003). Biochemical markers of bone metabolism in bone marrow edema syndrome of the hip. Bone.

[2] Crotti C, Watts NB, De Santis M, Ceribelli A, Fabbriciani G (2018). Acute phase reactions after zoledronic acid infusion: protective role of 25-hydroxyvitamin D and previous oral bisphosphonate therapy. Endocr Pract..

[3] Eriksen EF (2015). Treatment of bone marrow lesions (bone marrow edema). BoneKEy Rep.

[4] Flores-Robles BJ, Sanz-Sanz J, Sanabria-Sanchinel AA, Huntley-Pascual D, Andréu Sánchez JL (2017). Zoledronic acid treatment in primary bone marrow edema syndrome. J Pain Palliat Care Pharmacother.

[5] Horas K, Fraissler L, Maier G, Jakob F, Seefried L, Konrads C, Rudert M, Walcher M (2017). High prevalence of vitamin D deficiency in patients with bone marrow edema syndrome of the foot and ankle. Foot Ankle Int.

[6] Kavanagh KL, Guo K, Dunford JE, Wu X, Knapp S, Ebetino FH, Rogers MJ, Russell RGG, Oppermann U (2006). The molecular mechanism of nitrogen-containing bisphosphonates as antiosteoporosis drugs. Med Sci.

[7] Kon E, Ronga M, Filardo G, Farr J, Madry H, Milano G, Andriolo L, Shabshin N (2016). Bone marrow lesions and subchondral bone pathology of the knee. Knee Surg Sports Traumatol Arthrosc.

[8] Laslett LL, Doré DA, Quinn SJ, Boon P, Ryan E, Winzenberg TM, Jones G (2012). Zoledronic acid reduces knee pain and bone marrow lesions over 1 year: a randomised controlled trial. Ann Rheum Dis.

[9] Marcacci M, Andriolo L, Kon E, Shabshin N, Filardo G (2016). Aetiology and pathogenesis of bone marrow lesions and osteonecrosis of the knee. EFORT Open Rev.

[10] Meier C, Kraenzlin C, Friederich NF, Wischer T, Grize L, Meier CR, Kraenzlin ME (2014). Effect of ibandronate on spontaneous osteonecrosis of the knee: a randomized, double-blind, placebo-controlled trial. Osteoporos Int.

[11] O’Connell NE, Wand BM, McAuley J, Marston L, Moseley GL (2013). Interventions for treating pain and disability in adults with complex regional pain syndrome. Cochrane Database Syst Rev.

[12] Oehler N, Mussawy H, Schmidt T, Rolvien T, Barvencik F (2018). Identification of vitamin D and other bone metabolism parameters as risk factors for primary bone marrow oedema syndrome. BMC Musculoskelet Disord.

[13] Peterfy CG, Guermazi A, Zaim S, Tirman PFJ, Miaux Y, White D, Kothari M, Lu Y, Fye K, Zhao S, Genant HK (2004). Whole-organ magnetic resonance imaging score (WORMS) of the knee in osteoarthritis. Osteoarthr Cartil.

[14] Ringe JD, Brody J-J (2007). Review a review of bone pain relief with ibandronate and other bisphosphonates in disorders of increased bone turnover. Clin Exp Rheumatol.

[15] Ringe JD, Dorst A, Faber H (2005). Effective and rapid treatment of painful localized transient osteoporosis (bone marrow edema) with intravenous ibandronate. Osteoporos Int.

[16] Rolvien T, Schmidt T, Butscheidt S, Amling M, Barvencik F (2017). Denosumab is effective in the treatment of bone marrow oedema syndrome. Injury.

[17] Rondeau J-M, Bitsch F, Bourgier E, Geiser M, Hemmig R, Kroemer M, Lehmann S, Ramage P, Rieffel S, Strauss A, Green JR, Jahnke W (2006). Structural basis for the exceptional in vivo efficacy of bisphosphonate drugs. ChemMedChem.

[18] Simon MJK, Barvencik F, Luttke M, Amling M, Mueller-Wohlfahrt H-W, Ueblacker P (2014). Intravenous bisphosphonates and vitamin D in the treatment of bone marrow oedema in professional athletes. Injury.

[19] Sprinchorn AE, O’Sullivan R, Beischer AD (2011). Transient bone marrow edema of the foot and ankle and its association with reduced systemic bone mineral density. Foot Ankle Int.

[20] Thiryayi WA, Thiryayi SA, Freemont AJ (2008). Histopathological perspective on bone marrow oedema, reactive bone change and haemorrhage. Eur J Radiol.

[21] Vasikaran S, Eastell R, Bruyère O, Foldes AJ, Garnero P, Griesmacher A, McClung M, Morris HA, Silverman S, Trenti T, Wahl DA, Cooper C, Kanis JA (2011). Markers of bone turnover for the prediction of fracture risk and monitoring of osteoporosis treatment: a need for international reference standards. Osteoporos Int.

